# Interleukin 12-containing influenza virus-like-particle vaccine elevate its protective activity against heterotypic influenza virus infection

**DOI:** 10.1016/j.heliyon.2020.e04543

**Published:** 2020-08-08

**Authors:** Kenichi Maegawa, Shigeo Sugita, Youta Arasaki, Reiko Nerome, Kuniaki Nerome

**Affiliations:** a The Institute of Biological Resources, 893-2, Nakayama, Nago-shi, Okinawa 905-0004, Japan; b Equine Research Institute, Japan Racing Association, 1400-4, Shiba, Shimotsuke-shi, Tochigi 329-0412, Japan

**Keywords:** Biotechnology, Microbiology, Virology, Influenza, Vaccination, Antibody, Viral disease, Microbial biotechnology, Vaccines, *Bombys mori*, Eri silkworm, Cytokine, Avian influenza

## Abstract

To produce monovalent and bivalent influenza vaccines composed of virus-like particles (VLPs) containing hemagglutinin (HA), we generated four recombinant Baculoviruses derived from *Bombyx mori* nuclear polyhedrosis virus (BmNPV) and *Autographa california* nuclear polyhedrosis virus (AcNPV). Monovalent Fukushima (A/tufted duck/Fukushima/16/2011 [H5N1]) (FkH5) and Anhui (A/Anhui/1/2013 [H7N9]) (AnH7) VLP influenza vaccines were produced in silkworm pupae infected with FkH5-BmNPV or AnH7-BmNPV. To produce a bivalent FkH5 and AnH7 vaccine, the pupae were simultaneously inoculated with FkH5-BmNPV and AnH7-BmNPV. Then, interleukin (IL)-containing bivalent vaccines were produced by Eri silkworm pupae following triple infection with FkH5-AcNPV, AnH7-AcNPV, and IL-12-AcNPV. Fluorescent antibody tests in Sf9 cells triple-infected with FkH5-AcNPV, AnH7-AcNPV, and IL-12-AcNPV showed coexpression of FkH5, AnH7, and IL-12 antigens, suggesting the presence of VLPs containing all three antigens. We then performed competitive hemagglutination inhibition (CHI) tests to calculate the VLP vaccine constituents. Inoculation with two recombinant viruses led to the production of bivalent vaccines containing very similar amounts of the H5 and H7 antigens, suggesting that our dual infection system can be used to produce bivalent VLP vaccines. Immunisation of mice with our developed monovalent and bivalent VLP vaccines induced the production of HI antibody, which protected against a sublethal dose of influenza virus. These IL-12-containing vaccines tended to display increased protection against hetero-subtype influenza viruses.

## 1 Introduction

Recent outbreaks of highly pathogenic avian influenza viruses have threatened both the poultry industry and human health [1, 2, 3]. In the first outbreak in 1997, 6 out of 18 avian influenza-infected patients died due to multiple organ failure [3, 4]. The characteristics of the isolated viral genomes indicated that all human infections occurred at chicken markets in Hong Kong where diseased chickens were introduced from epizootic poultry farms [3, 5].

Since then, avian influenza has attracted considerable public attention, and avian influenza vaccines have been developed using various technologies. These vaccines have been produced by various methods including: 1) using temperature-sensitive mutants [6], 2) cell culture [7], 3) hemagglutinin (HA)-containing virus-like particles (VLPs) [8, 9], and 4) DNA-based vaccines [10, 11]. With an aim to provide inexpensive, effective vaccines against avian influenza, we developed a recombinant baculovirus system for the large-scale production of H5 and H7 HA-containing VLP vaccines in silkworms [8, 12]. We also established a similar system in Eri silkworms [13] that allows the production of HA titres greater than 30 million per pupa. H7-containing VLP vaccines produced using different systems elicited strong immune responses in murine, ferret, and avian models [14, 15, 16, 17].

Multiple recent reports of zoonotic infections caused by H5N8 and H5N6 avian influenza viruses have raised concerns regarding the threat to avian and human health [18]. Recent H7 influenza virus infections in humans has also elevated the threat of infection in urban environments [19, 20, 21]. These epizootic and epidemic reports have led to the development of multivalent influenza vaccines [22, 23] and active vaccination programmes worldwide [24].

To prevent influenza virus infections in humans and poultry, we have implemented the following strategies. First, we attempted to effectively and rapidly produce inexpensive bivalent and multivalent vaccines in silkworms on a large scale to provide high-quality vaccines for the poultry and healthcare industries. Then, we inserted membrane-anchored interleukin (IL)-12 into VLP vaccines to enhance mucosal immune responses and elicit cellular immune responses [25, 26, 27, 28]. Khan et al. [25] demonstrated that coexpression of membrane-anchored IL-12 and IL-23 with influenza vaccines resulted in enhanced mucosal immune responses. Here, we describe an effective multiple-infection system that can be used to synthesise bivalent or VLP vaccines containing IL-12 and multiple HA VLP antigens.

## 2 Materials and methods

### 2.1 Cells and viruses

*Bombyx mori* Bm-N cells and *Spodoptera frugiperda* ovarian Sf9 cells (Sigma Aldrich, Tokyo, Japan) were cultured in Grace's insect medium (Thermo Fisher Scientific) supplemented with 10% foetal bovine serum (FBS). Madin-Durby canine kidney (MDCK) cells were cultured in Eagle's minimum essential medium (MEM) containing 10% FBS.

The P6E strain of *Bombyx mori* nuclear polyhedrosis virus (BmNPV) was used to generate FkH5-BmNPV and AnH7-BmNPV recombinant viruses. *Autographa californica* nuclear polyhedrosis virus (AcNPV) was used to produce FkH5-AcNPV and AnH7-AcNPV recombinant viruses.

Influenza virus A/PR/8/34 (H1N1) strain PRH1 was cultivated in MDCK cells. Recombinant influenza viruses HkH5 (RG-A/BarnSwallow/Hong Kong/1161/2010-A/PR/8/34 [R][6 + 2] [H5N1]) and AnH7 (RG-A/Anhui/1/2013-A/PR/8/34[R][6 + 2] [H7N9]), were kindly provided by Dr. R.G. Webster (St. Jude Children's Research Hospital, Memphis, TN, USA). Viruses were propagated in 11-day-old embryonic chicken eggs and MDCK cells.

### 2.2 Generation of recombinant viruses

The FkH5 and AnH7 genes were synthesised by GENEWIZ (Saitama, Japan). The methods used to generate BmNPV- and AcNPV-based recombinant viruses containing these HA genes have been described previously [8, 12].

**Figure 1:**
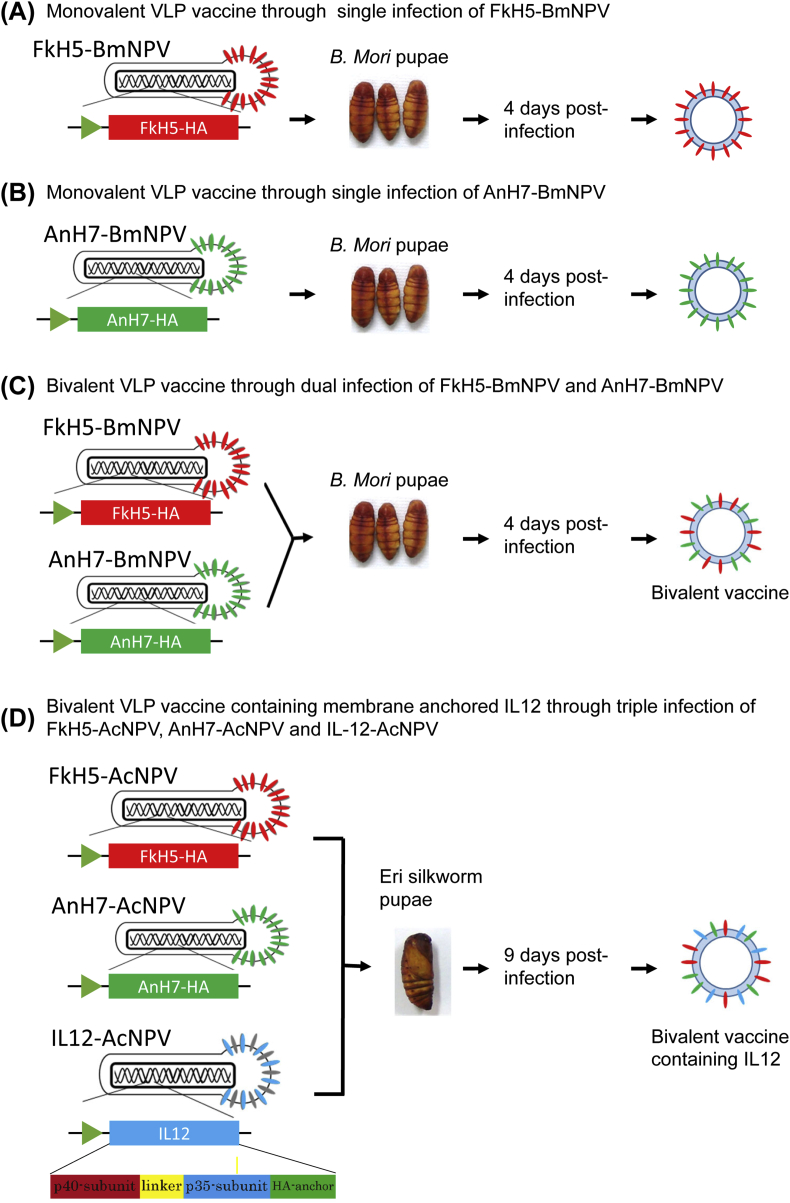
Production of VLP vaccines from silkworm pupae infected with recombinant Baculoviruses. Monovalent vaccines were produced from *Bombyx mori* pupae infected with (A) FkH5-BmNPV- and (B) AnH7-BmNPV. To produce bivalent FkH5+AnH7-VLP vaccines, *B. mori* pupae were co-infected with FkH5-Bm NPV and AnH7-Bm NPV, and bivalent vaccines were extracted from the infected pupae (C). Bivalent vaccines containing membrane-anchored IL-12 were produced by Eri silkworm pupae triple infected with IL-12-AcNPV, FkH5-AcNPV, and AnH7-AcNPV (D).

The IL-12–40/35 genes were also synthesised by GENEWIZ. Briefly, IL-12–40/35 was constructed by combining two IL-12 subunits (p40 and p35) with a glycine linker (L1) such that p40 was located at the N-terminus, and the membrane anchor region, which consists of the influenza strain WSN (H1N1) HA membrane-anchoring domain (Figure 1D), was located in the C-terminus [25]. Then, the IL-12–40/35 gene was inserted downstream of the pFastBac1 polyhedrin promotor. The generated plasmids were transformed into DH10Bac cells. Subsequently, recombinant Ac-NPV DNA was purified and transfected into Sf9 cells as described previously [13]. After 1 week of culture, the supernatant, containing recombinant Ac-NPV viruses, was harvested and used in subsequent experiments.

### 2.3 Production of VLP vaccines in silkworm pupae

*B. mori* and Eri silkworm pupae were used to produce VLP vaccines by infecting *the* silkworms with BmNPV and AcNPV recombinant viruses, respectively (Figure 1). Baculovirus inoculation and preparation of VLP vaccines were performed as previously described [13].

### 2.4 Hemagglutination and hemagglutination inhibition (HI) tests

Hemagglutination and HI tests were performed as described previously [8, 12].

### 2.5 Competitive HI (CHI) tests to calculate HA antigen content in the bivalent vaccines

CHI tests were performed to calculate the HA antigen content in the produced multivalent VLP vaccines. Briefly, 25 μl of monovalent FkH5-VLP vaccine or AnH7-VLP vaccine were mixed with 0, 5, 10, 15, 20, 25, 30, or 35 μl of homologous anti-FkH5 serum or anti-AnH7 serum, and PBS was added to bring the final volume to 60 μl. After mixing, the tubes were incubated for 30 min at room temperature (20–25 °C), and the HA titres were determined.

**Figure 2:**
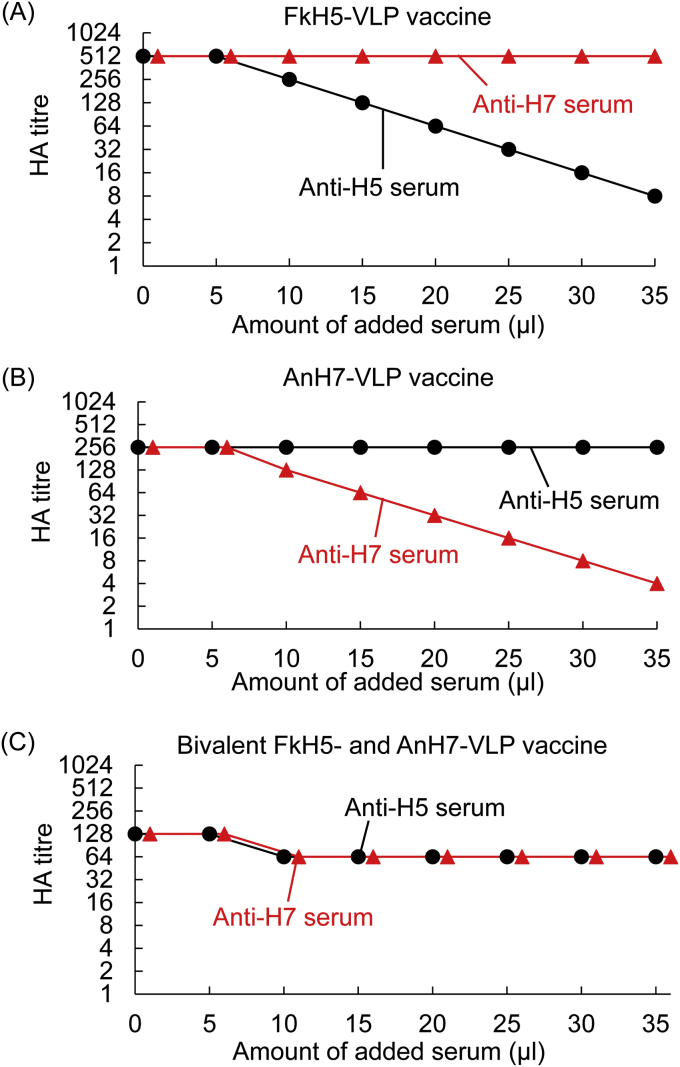
CHI tests using anti-FkH5HA and anti-AnH7HA sera. Monovalent FkH5-VLP vaccine (A) and AnH7-VLP vaccine (B) and bivalent FkH5+AnH7-VLP vaccine (C) were evaluated by CHI tests using anti-FkH5HA and anti-AnH7HA sera.

Although the addition of 5 μl of serum did not affect the HA titre, the addition of 10 μl or more of homologous anti-FkH5 serum to the FkH5-VLP vaccine resulted in a linear reduction in the HA titre on a semilogarithmic plot (Figure 2A). In contrast, anti-AnH7 serum did not affect the titre. When both anti-FkH5 and anti-AnH7 sera were added to the AnH7-VLP vaccine, only the anti-AnH7 serum affected the HA titre, which was linearly reduced in the presence of 10 μl or more of the serum (Figure 2B). These results indicated that the anti-FkH5 and anti-AnH7 antibodies are not cross-reactive. Therefore, when anti-FkH5 serum is added to bivalent FkH5+AnH7 vaccines, the residual HA titre should correspond to the AnH7 HA antigen titre. Conversely, the FkH5 antigen HA titre can be similarly calculated when anti-AnH7 antibody is added. Therefore, the H5 and H7 antigen contents could be determined using this CHI test. The residual HA titre, as determined in the presence of anti-FkH5 and anti-AnH7 sera was 64 for both antigens (Figure 2C). Therefore, the bivalent vaccine contains the same amounts of H5 and H7 antigens.

### 2.6 Hemadsorption tests

Bm-N and Sf9 cells were infected with recombinant viruses. Then, the cells were washed once with PBS and used in hemadsorption tests with 0.5% chicken erythrocytes, as described previously [12].

### 2.7 Fluorescent antibody (FA) tests

Bm-N and Sf9 cells were infected with recombinant viruses and incubated in Grace's insect medium supplemented with 10% FBS at 27 °C for 3 days. Then, FA tests were performed as described previously [8, 12].

Antisera against H1HA, H5HA, and H7HA were prepared from mice immunised with purified PRHA, HkH5, and AnH7 influenza viruses (HA titres: 2,048–4,096), respectively. Goat anti-IL-12 polyclonal antibody was purchased from Peprotech (USA). The following secondary antibodies were used: Alexa Fluor 488-conjugated goat anti-mouse IgG (Cell Signaling Technology, USA), Alexa Fluor 555-conjugated goat anti-rabbit IgG (Cell Signaling Technology), Alexa Fluor 555-conjugated donkey anti-goat IgG (Abcam, UK), and Alexa Fluor 488-conjugated donkey anti-mouse IgG (Abcam).

### 2.8 Analysis of the protective efficacy against intranasal challenge with influenza viruses

Five groups of 4-week-old female ddY mice purchased from Japan SLC co., Ltd. (Shizuoka, Japan) were immunised intraperitoneally with PBS, inactivated PRH1, and IL-12-containing FkH5- and AnH7-AcVLP vaccines. The VLP vaccines contained HA titres of 4,000–16,800. Two weeks later, the animals were immunised a second time as described above. Six weeks later, the mice were intranasally challenged with virulent PRH1, FkH5, and AnH7 viruses. Animal health and survival were observed daily for 10 days. Mouse experiments were carried out in specific pathogen-free (SPF) conditions, with the approval of the Animal Welfare and Animal Care Committee, including the Animal Ethics Committee of the Institute of Biological Resources, Okinawa, Japan.

## 3 Results

### 3.1 AcNPV-infected Sf9 cells produce recombinant vaccines

**Figure 3:**
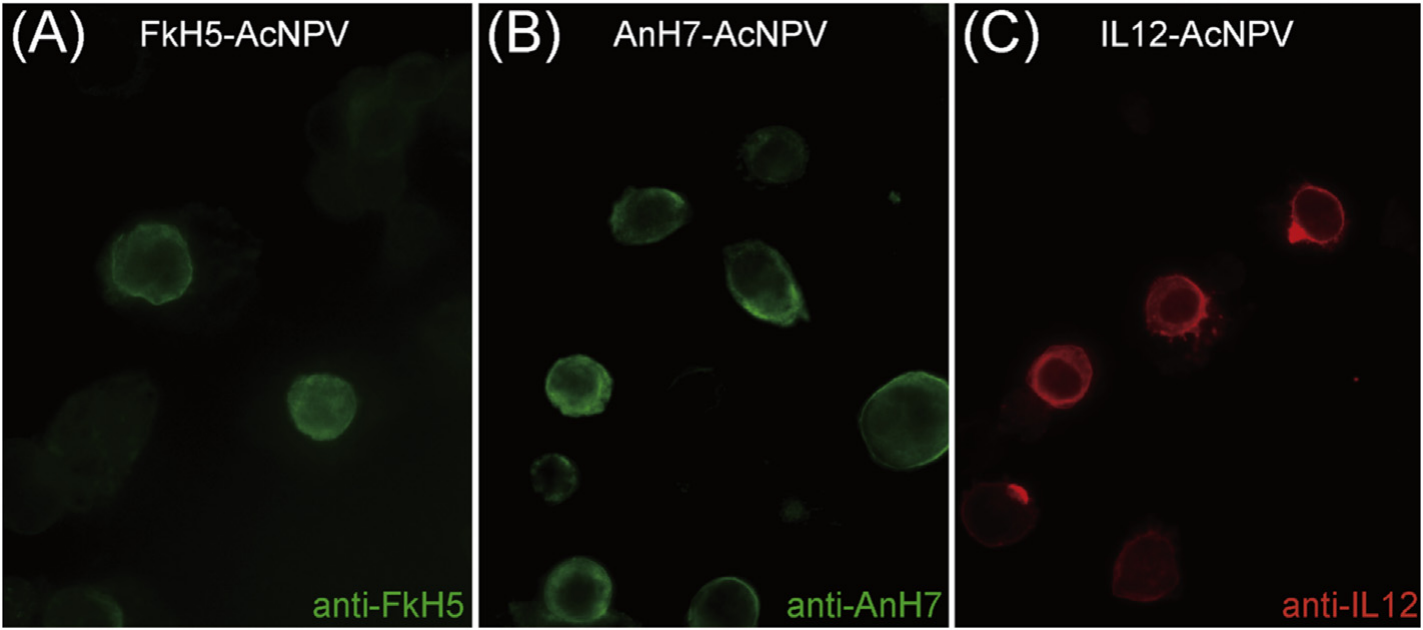
Expression of FkH5HA, AnH7HA, and IL-12 antigens in recombinant AcNPV-infected Sf9 cells. Sf9 cells were infected with FkH5-AcNPV (A), AnH7-AcNPV (B), and IL-12-AcNPV (C). Then, the infected cells were fixed with methanol and stained with anti-FkH5 mouse serum (A), anti-AnH7 mouse serum (B), or anti-IL-12 goat polyclonal antibody (C). Antibody staining was detected with Alexa Fluor 488-conjugated anti-mouse IgG (A, B) and Alexa Fluor 555-conjugated anti-goat IgG (C).

We previously established a recombinant AcNPV system in Eri silkworms that produces abundant recombinant proteins in pupae [13]. In this study, we generated three recombinant AcNPV viruses: FkH5-AcNPV, AnH7-AcNPV, and IL-12-AcNPV. FkH5-AcNPV and AnH7-AcNPV have been described previously [13], and they respectively produced H5- and H7-subtype influenza virus HA proteins (Figure 3A, B). IL-12-AcNPV produced membrane-anchored, single-chain IL-12, which was described previously [25]. Membrane-anchored IL-12 is an immunopotentiating agent for influenza vaccines; therefore, the efficacy of VLP vaccines should be improved by introducing IL-12. IL-12 protein production was confirmed by FA tests (Figure 3C). Almost all the Sf9 cells infected with FkH5-AcNPV, AnH7-AcNPV, or IL-12-AcNPV were positively stained with anti-H5HA serum, anti-H7HA serum, or anti-IL-12 antibody, respectively. Next, we attempted to produce multivalent VLP influenza vaccines using these recombinant viruses (Figure 3).

### 3.2 Sf9 cells infected with multiple recombinant AcNPVs coexpress HA and IL-12

**Figure 4:**
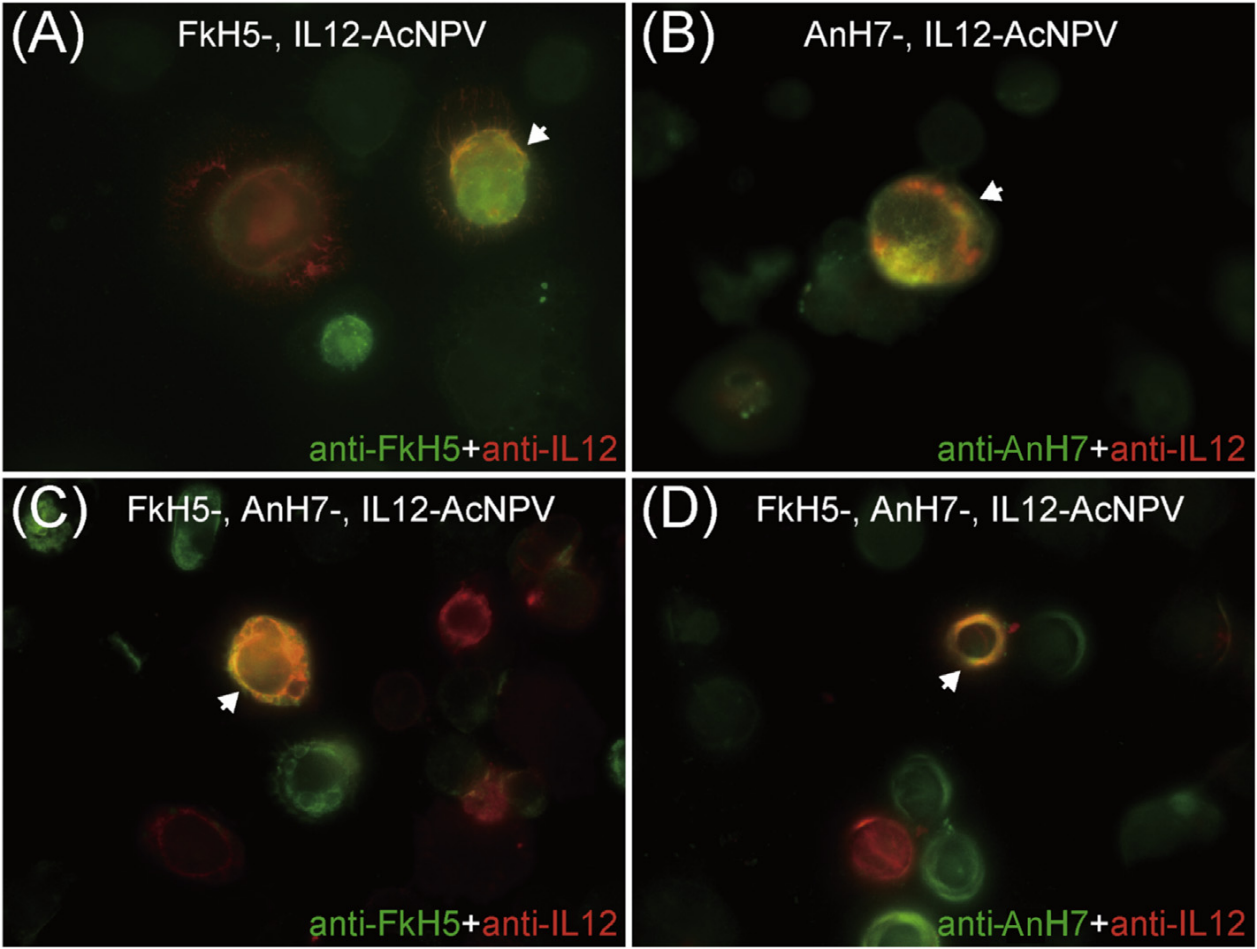
Confirmation of FkH5, AnH7, and IL-12 antigen expression in triple-infected Sf9 cells. Sf9 cells were co-infected with FkH5-AcNPV and IL-12-AcNPV (A), AnH7-AcNPV and IL-12-AcNPV (B), or FkH5-AcNPV, AnH7-AcNPV, and IL-12-AcNPV (C, D). Infected cells were fixed with methanol and stained with anti-IL-12 goat polyclonal antibody (A, B, C, D), anti-FkH5 mouse serum (A, C), or anti-AnH7 mouse serum (B, D). Stained cells were then labelled with Alexa Fluor 488-conjugated anti-mouse IgG and Alexa Fluor 555-conjugated anti-goat IgG. Double-positive cells expressing two antigens are indicated by arrows.

Since the goal of this study was to produce VLP vaccines containing multiple antigens by co-infecting multiple recombinant AcNPVs, it was important to examine the expression of these antigens. To address this, Sf9 cells were simultaneously infected with multiple recombinant viruses and analysed in FA tests. Sf9 cells were dually infected with IL-12-AcNPV and either FkH5-AcNPV or AnH7-AcNPV, and then co-expression of both IL-12 and HA was detected by antibody co-labelling (Figure 4A, B). The results clearly demonstrated the coexpression of multiple antigens and the feasibility of multiple-antigen production by VLP vaccines using a co-infection method.

We hypothesised that IL-12-containing VLP vaccines could be produced in silkworm pupae by triple infection with FkH5-AcNPV, AnH7-AcNPV, and IL-12-AcNPV. Because dual antigen expression was observed in dual-infected cells, we next tried to examine whether triple-infected cells expressed IL-12 and dual HA antigens. Indeed, when Sf9 cells were triple infected with IL-12-AcNPV, FkH5-AcNPV, and AnH7-AcNPV, both HA antigens (H5 and H7) and IL-12 were expressed (Figure 4C, D).

### 3.3 Hemadsorbing activity of recombinant, co-infected Sf9 cells

**Figure 5:**
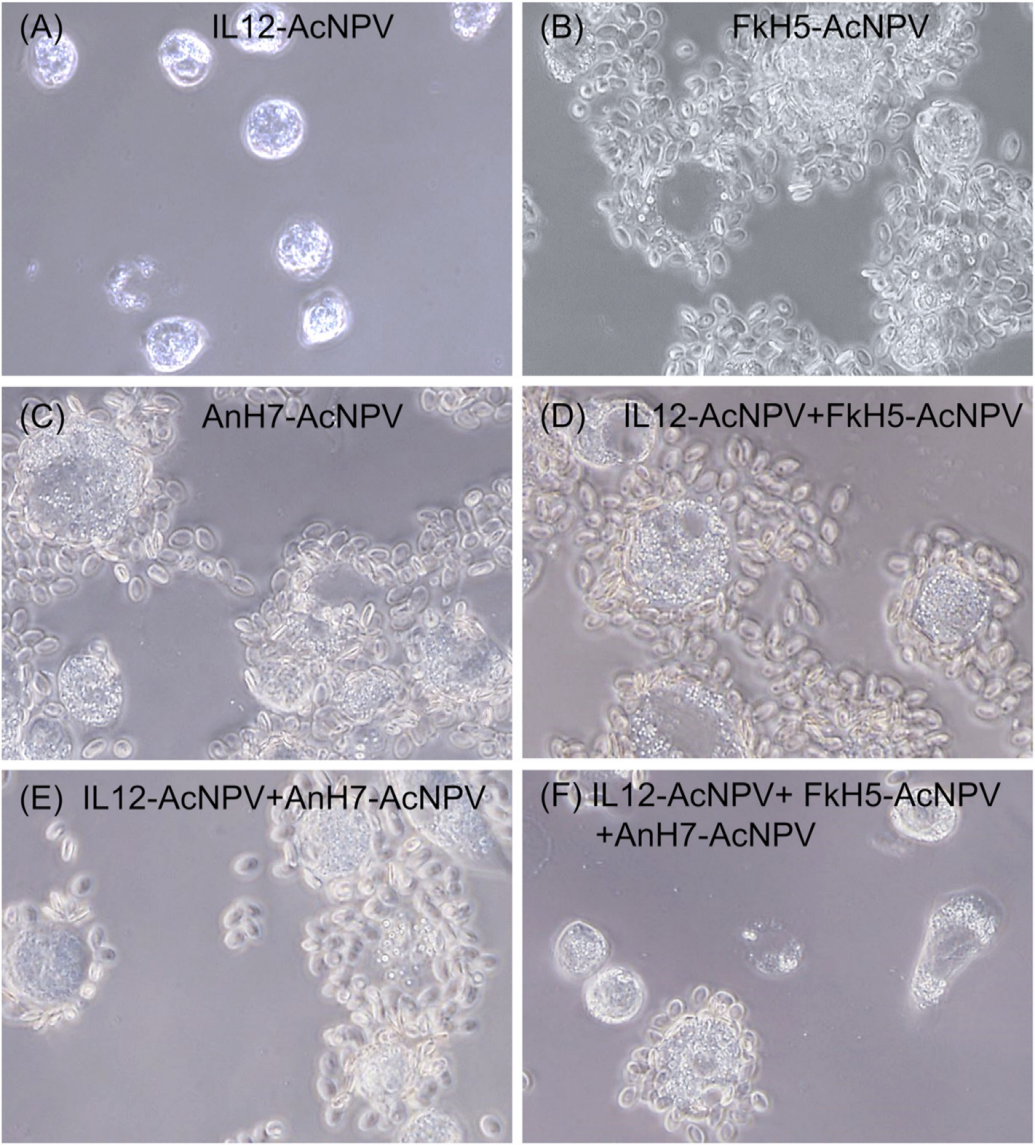
Confirmation of VLP HA antigens on the surface of Sf9 cells infected with recombinant Baculoviruses. Sf9 cells were infected with IL-12-AcNPV (A, D, E, F), FkH5-AcNPV (B, D, F), or AnH7-AcNPV (C, E, F). Then, the hemadsorption activity of the infected cells was examined.

To confirm cell surface localisation of the HA antigens in cells infected with recombinant AcNPV, hemadsorption activity was assessed (Figure 5). Hemadsorption assays can be used to verify the biological completeness of the HA protein on the surface of recombinant virus-infected cells, because erythrocytes are added external to the infected cells.

No erythrocyte adsorption was observed using IL-12-AcNPV-infected Sf9 cells (Figure 5A). In contrast, clear hemadsorption was detected using FkH5-AcNPV- and AnH7-AcNP-infected Sf9 cells, regardless of the presence of IL-12-AcNPV (Figure 5B–F), indicating the presence of the FkH5 and AnH7 antigens on the cell surface. Similar hemadsorption was observed for IL-12-AcNPV co-infected cells, suggesting that membrane-anchored IL-12 does not interfere with the transport of H5 or H7 HA to the cell surface.

### 3.4 Large-scale production of mono- and bivalent vaccines

**Figure 6:**
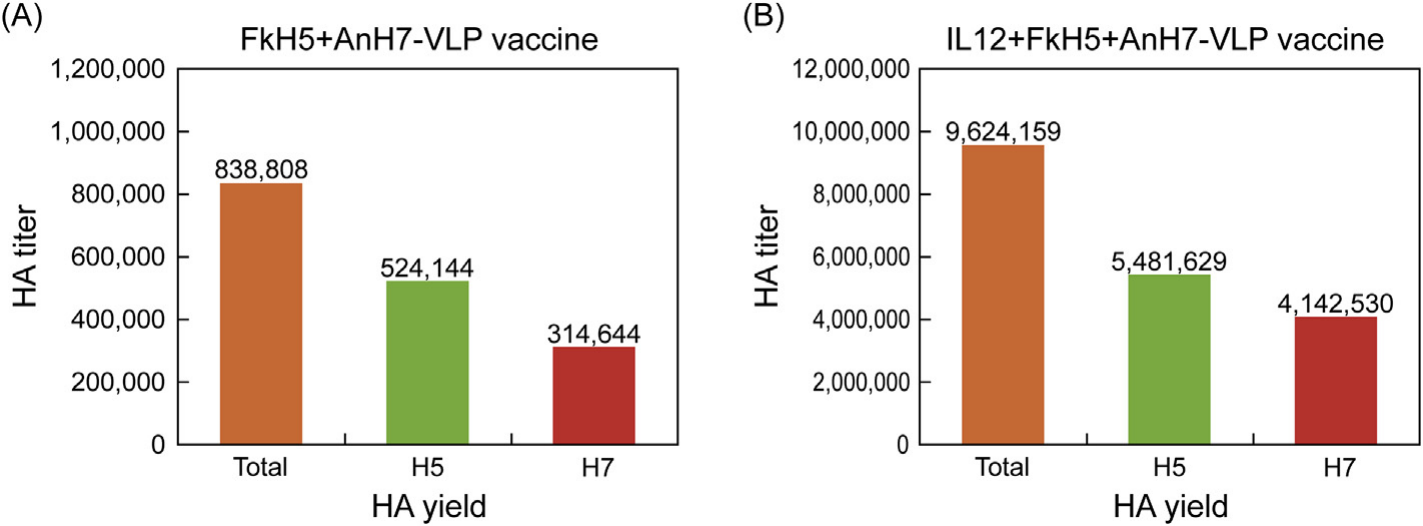
Determination of antigen content by CHI test. (A) Twenty *B. mori* pupae co-infected with FkH5-BmNPV and AnH7-BmNPV yielded 12 mL of bivalent VLP vaccine (HA titre: 838,808). CHI tests showed that the bivalent vaccine contained HA titres of 524,144 for H5 antigen and 314,664 for H7 antigen. (B) Ten *B. mori* pupae triple-infected with IL-12-AcNPV, FkH5-AcNPV, and AnH7-AcNPV yielded 5 mL of IL-12-containing bivalent VLP vaccine with an HA titre of 9,624,159. CHI tests showed that the bivalent vaccine contained titres of 5,481,629 for H5 antigen and 4,142,530 for H7 antigen.

As reported previously, we successfully and separately produced large amounts of the FkH5- and AnH7-VLP vaccines using *B. mori* pupae infected with FkH5-BmNPV and AnH7-BmNPV, respectively [13]. Using these two recombinant viruses, we attempted to produce bivalent VLP vaccines containing both FkH5 and AnH7 via dual infection of *B. mori* pupae. To assess co-expression of the two HA antigens, co-infected pupae were homogenised in PBS containing 0.5% sodium thiosulfate as an antioxidant. Then, the homogenate was purified by sucrose gradient centrifugation, as described previously [12]. The isolated FkH5+AnH7-VLPs were used as a bivalent vaccine. We then quantified the H5 and H7 HA antigens in the bivalent vaccine using a simple CHI test. The isolated bivalent vaccine had an HA titre of 838,808, including 62% H5 antigen (H5 HA titre: 524,144) and 38% H7 antigen (H7 HA titre: 314,644; Figure 6A).

Finally, we attempted to produce bivalent vaccine containing membrane-anchored IL-12 by triple infection of Eri silkworm pupae with FkH5-AcNPV, AnH7-AcNPV, and IL-12-AcNPV. The triple-infected pupae were homogenised in PBS containing 0.5% sodium thiosulfate, and the antigens were purified by sucrose density gradient centrifugation. CHI tests showed that the IL-12-containing VLP bivalent vaccine contained an HA titre of 9,624,159 (Figure 6B), with 57% FkH5 (5,481629) and 43% AnH7 (4,142,530). These results indicate that the VLP antigen yield in Eri silkworms was greater than that in *B. mori* silkworms as reported previously [13].

Large-scale production of bivalent vaccines and subsequent analyses indicated that: 1) large amounts of bivalent H5–H7 VLP vaccines containing nearly equivalent levels of both HA antigens can be easily obtained through dual or triple infection of silkworm pupae by adjusting the MOI, and 2) CHI tests can clearly distinguish the different HA antigens in bivalent vaccines. These results suggest that CHI tests might be useful for antigenic differentiation of other bivalent, trivalent, or multivalent vaccines.

### 3.5 Antibody production in mice immunised with mono- or divalent VLP vaccines

**Figure 7:**
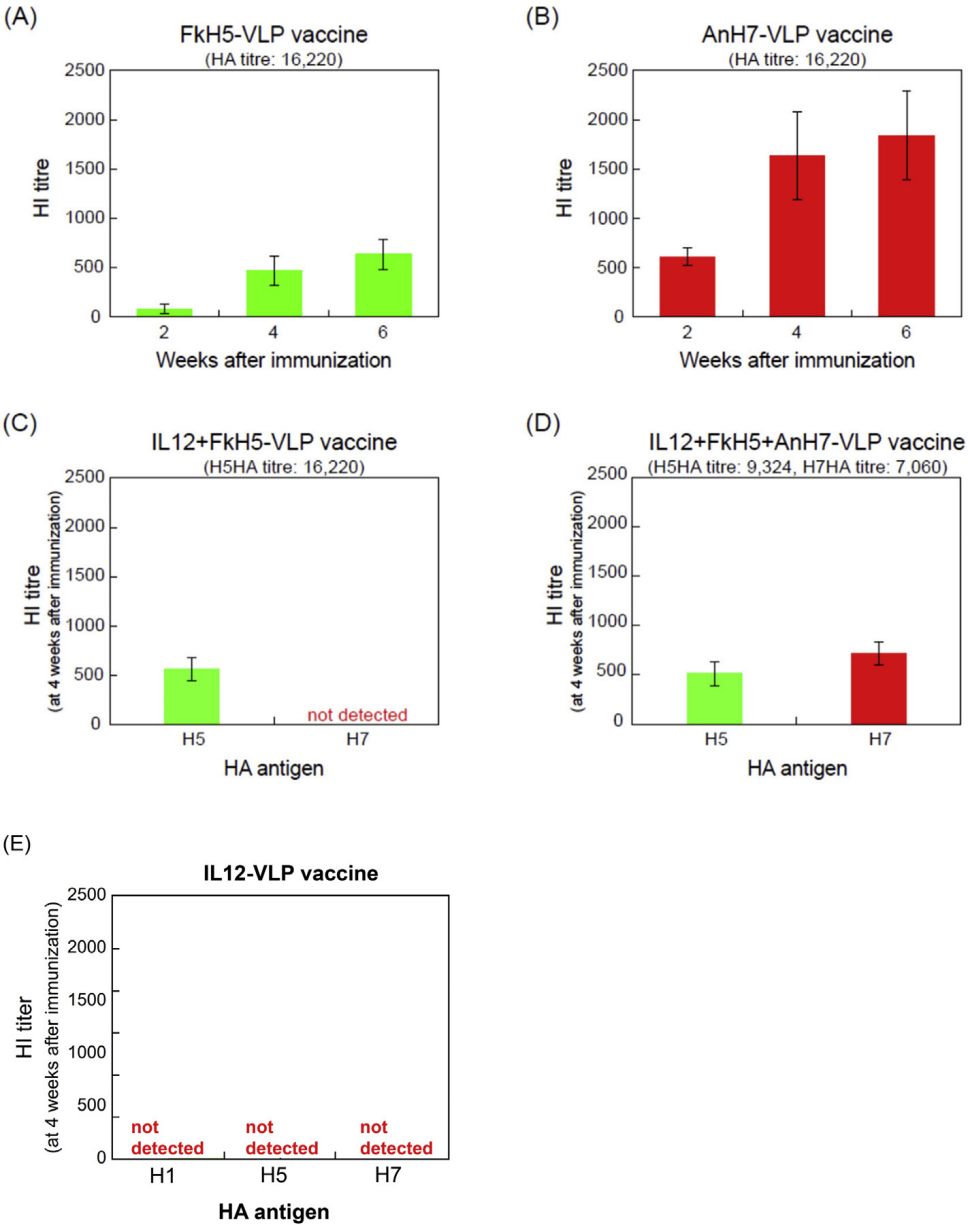
Evaluation of immune responses by assessment of HI antibody production induced by VLP vaccines. Time course of the changes in the HI titres in the sera of mice (n = 5 per group) immunised with monovalent FkH5-VLP (A) and AnH7-VLP (B) vaccines. HI antibody production at 4 weeks after immunisation with IL-12-containing FkH5-VLP vaccine (C) or IL-12-containing FkH5+AnH7-VLP vaccine (D). HI antibody production in mice immunised with an IL-12-VLP vaccine (E). Data represent the means ± SEM.

To examine the immunogenicity of the developed VLP vaccines, we measured HI titres in the sera of mice immunised with the vaccines. Vaccination with FkH5-VLP or AnH7-VLP led to an increase in the serum HI titre. The HI titres in FkH5-VLP-vaccinated animals were 83, 467, and 640 at 2, 4, and 6 weeks post-immunisation, respectively (Figure 7A), while those in AnH7-VLP-vaccinated mice were 614, 1638, and 1843 at 2, 4, and 6 weeks post-immunisation, respectively (Figure 7B). Mice immunised with the AnH7-VLP vaccine showed 3–7 times higher HI titres than mice immunised with the FkH5-VLP vaccine, although the amounts of antigens used for immunisation were the same (HA titre: 16,220).

Although IL-12-containing VLP vaccines are easily produced via multiple infection, the immunogenicity of these vaccines might be different from that of vaccines prepared from singly infected pupae. Although IL-12 was included in the vaccine, its adjuvant effect was not determined in this study. However, mice immunised with the FkH5-VLP vaccine exhibited an H5 HI titre of 467 at 4 weeks post-immunisation, which was similar to that of mice immunisation with the IL-12-containing FkH5 vaccine (Figure 7A–C).

Mice immunised with a bivalent vaccine possessing containing HA titres of 9,324 (FkH5) and 7,060 (AnH7) had similar HI titres against HkH5 and AnH7 viruses, respectively (Figure 7D). 247–248: Additionally, in a control experiment, mice immunised with IL-12 alone did not produce FkH5 and AnH7 antigens (Figure 7E).

### 3.6 Evaluation of the protective efficacy of IL-12-VLP vaccines

**Figure 8:**
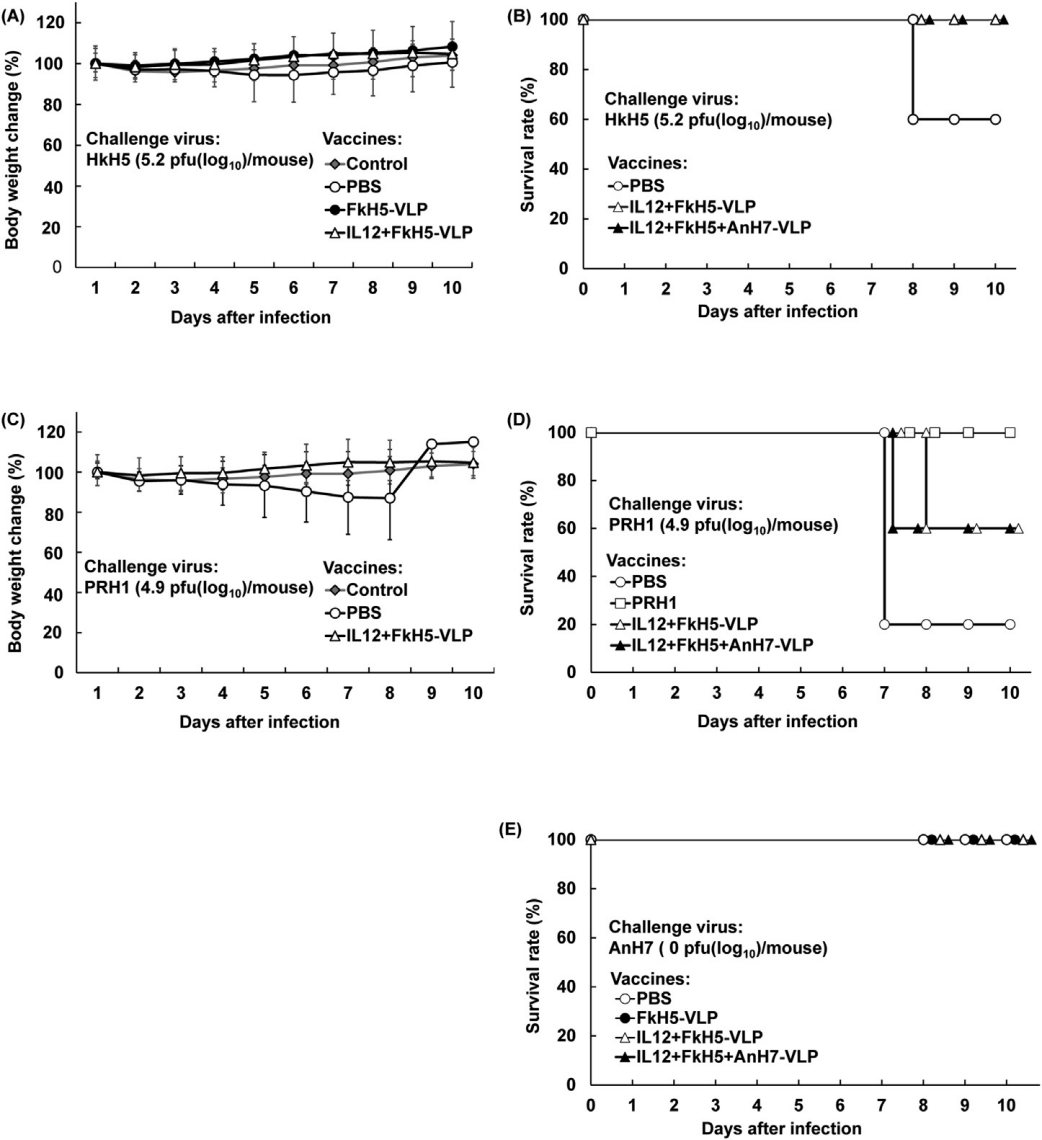
Evaluation of the protective effect of different VLP vaccines in mice. (A) Change (%) of body weight in control mice and test mice immunised with PBS, FkH5-VLP and IL12 + FkH5-VLP vaccines. (B) Survival rate of mice immunised with PBS, IL12 + FkH5-VLP, or IL12 + FkH5+AnH7-VLP vaccines following infection with HkH5 virus. (C) Body weight changes (%) in control and immunised with PBS, IL12 + FkH5-VLP vaccine following infection with PRH1 virus. (D) Survival rates of mice immunised with four different vaccines and subsequently with PRH1 virus infected mouse group. (E) Tentative evaluation of each VLP vaccine against AnH7 virus infection, but failed its evaluation due to non-growth characteristic of a recombinant An H7 virus.

Antibody production with different VLP vaccines led us to evaluate the VLP vaccines for protective efficacy against challenge influenza viruses. So, we immunised three groups comprising of 5 mice with either PBS, IL12 + FkH5-VLP vaccine, or IL12 + FkH5+AnH7-VLP vaccine including one unimmunized control. Since the change of body weight is an important index for evaluating the progression of pneumonia of test mouse, we first examined the body weight of test mice, and the results are shown in Figure. 8A–C. Resultant body weights of each mouse group comprising of unimmunised and non-challenged group increased constantly during 10 days (Figure 8A). In contrast, a slight decrease of body weight was determined in the mice immunised with PBS and challenged with the HkH5 virus until 5 days after infection. Interestingly, the two groups of mice immunised with either FKH5-VLP or IL-12 + FkH5 VLP vaccine did not show any reduction in body weights after infection with HkH5 (Figure 8A). However, although a slight decrease in body weight was seen in FkH5-VLP vaccinated group, this tendency recovered after 7 days. It was evident that the changes of body weight of test mice immunised with IL-12 + FkH5 VLP were not influenced with H5 virus infection (Figure 8A). In a same experiment, unimmunised and PRH1 virus-infected mouse group showed an obvious decrease of body weight, suggesting the occurrence of severe pneumonia (Figure 8C). This result led us to the following experiment using different VLP vaccines. Thus, we immunised three groups with PBS, IL12 + FkH5-VLP vaccine and IL12 + FkH5+AnH7-VLP vaccine. After 6 weeks, the above test groups were challenged with HkH5 virus. As a result, in unimmunised control mice, two in unimmunized group were found to be died at 8 days after infection (Figure 8B). It was a sharp contrast that all mice immunised with IL12 containing both vaccines survived during 10 days-entire test period. This result showed that both monovalent and divalent VLP vaccines containing IL12 induce protective immunity against H5 influenza virus (Figure 8B). When compared the above result, it was of interest to examine the protective activity of VLP vaccines against antigenically heterologous influenza virus. In next experiment, four groups were immunised with PBS (negative control), inactivated whole PRH1 virus vaccine (positive control), IL12-containing FkH5-VLP and IL12 containing FkH5- and AnH7-VLP vaccines. And 6 weeks latter, all mouse groups were intranasally infected with PRH1 virus. As a result, dead mice were not confirmed in any group until 6 days after challenge infection (Figure 8D). However, at 7 days after infection, four mice were found to be dead in PBS control group (80% mortality and 20% survival). In contrast, all mice immunised with inactivated PRH1 vaccine survived during entire test period. However, 2 mice immunised with FkH5 and AnH7 vaccines were dead at 7 days after infection and subsequently, further 2 mice in the FkH5-VLP group died at 8 days. These results indicated that IL12 containing VLP vaccine partly elevate protective activity against even distinct HA subtype of influenza A virus. However, as shown in Figure 8E, we did not evaluate protective activity of these different VLP vaccines against AnH7 virus due to failure of growth of AnH7 virus in mouse lung (Figure 8E).

## 4 Discussion

We developed a method for large-scale production of divalent vaccines containing IL-12. Vaccines produced using this method may reduce the threat of highly pathogenic avian influenza virus infection in birds and humans. Although new influenza viruses may appear in the future, our system should allow the rapid production of strain-specific vaccines against these viruses.

One of greatest communicable disease threats is a pandemic outbreak of avian influenza, which includes infections caused by the H5, H7, and H9 strains. Indeed, the World Bank recently reported an economic loss of $5.5 trillion due to the 2005 pandemics in developed countries (Japanese newspaper, Ryukyu Simpo, p.3, 14 May 2017). Recent influenza outbreaks caused by avian H7N9 have occurred over all the world [21]. In winter 2016, an outbreak of a virulent avian H7N6 strain in northern Japan led to the killing of 340,000 chickens in Aomori and Niigata to prevent further outbreaks. As preventative measures, 2,750,000 non-diseased chickens were killed in Japan from 2004 to 2014 (Japanese newspaper, Asahi Shimbun, p.28, 29 November 2015). Nation-wide influenza vaccination programmes are used in several countries to protect chickens [22]. Vaccine coverage is particularly high in China, Egypt, Hong Kong, Mongolia, and Vietnam. Widespread vaccination and the development of newer vaccines may prevent influenza outbreaks in human populations and protect several bird species.

To improve the response to new influenza strains, we have developed new methods by focusing on the following: 1) providing large amounts of avian influenza vaccines to many parts of the world, including low- and middle-income countries; 2) developing highly effective vaccines that protect bird species, including chickens; 3) developing and mass-producing effective, inexpensive vaccines; and 4) developing a complete, multivalent vaccine production system prior to the appearance of new viruses transmitted to humans from birds, using the 16 influenza A subtypes that are currently circulating in avian populations.

The monovalent and divalent vaccines and the methods developed in this study may satisfy these above conditions. Monovalent vaccines produced in silkworm pupae showed high yields (HA titre: 6,583,673 per pupa). Assuming 90% loss during purification, the resultant product should contain 658,367 HA per silkworm. Based on this yield and the administration of 5,000 HA per chicken, a single silkworm pupa should be able to treat 131 young adult chickens. Bivalent vaccines are highly potent, inexpensive vaccines that can be used in birds and humans. Bivalent vaccines with HA titres of several thousand could elicit the production of thousands of HI antibodies. Because of the low cost of production in silkworms, the antigenic quantities of these vaccines could be increased ten-fold, resulting in even higher HI antibody production.

It may be difficult to increase the protective immunity against some influenza A HA subtypes. Therefore, we conjugated IL-12 to H5 and H7 VLP antigens. These conjugated viruses elicited elevated HI antibody production. IL-12-containing bivalent H5 and H7 vaccines enhanced the survival of mice challenged with HkH5 and H1N1 subtype (PRH1) influenza. These results suggest that the introduction of certain cytokines into VLP vaccines may be a useful method for producing a universal vaccine, although this requires additional studies.

The introduction of cytokines into VLP vaccines is not limited to silkworm-derived VLP vaccines, as cytokines have been used in different influenza virus vaccines. For example, a trivalent H5, H7, and H9 vaccine produced from a single DNA plasmid with cytokines displayed greater protective efficacy [23, 24]. The results of this study represent a step closer towards enhanced vaccine development.

As described in previously [26], co-immunisation with IL-12 cDNA and *Coccidioides* protective antigen cDNA enhanced immunity against *Coccidioides immitis*. The effect of IL-2 and granulocyte macrophage colony-stimulating factor (GM-CSF) on the efficacy of influenza vaccines was investigated previously [27]. In addition, IL-23 is known to play a role in mucosal immunity [25], and adult mice mucosally immunised with IL-12- and IL-23-containing influenza vaccines produced virus-specific nasal IgA, leading to reduced viral titres in lung tissue after challenge. Cytokines such as IL-1-6, IL-12, and IL-15 show promise as vaccine adjuvants [27], because they may increase the rates of survival against different virus infections. Therefore, we suggest combining these cytokines with VLP influenza vaccines in future vaccine development.

## Declarations

### Author contribution statement

Kenichi Maegawa, Shigeo Sugita, Youta Arasaki: Performed the experiments; Contributed reagents, materials, analysis tools or data.

Reiko Nerome: Analyzed and interpreted the data; Contributed reagents, materials, analysis tools or data.

Kuniaki Nerome: Conceived and designed the experiments; Performed the experiments; Analyzed and interpreted the data; Wrote the paper.

### Funding statement

This research did not receive any specific grant from funding agencies in the public, commercial, or not-for-profit sectors.

### Competing interest statement

The authors declare no conflicts of interest.

### Additional information

No additional information is available for this paper.
